# A Novel Porous Butyryl Chitin–Animal Derived Hydroxyapatite Composite Scaffold for Cranial Bone Defect Repair

**DOI:** 10.3390/ijms24108519

**Published:** 2023-05-10

**Authors:** Wei Zhang, Zhiwen Jiang, Jinhua Chi, Huanchao Sun, Hongjian Li, Wanshun Liu, Baoqin Han

**Affiliations:** 1 Laboratory of Biochemistry and Biomedical Materials, College of Marine Life Sciences, Ocean University of China, Qingdao 266003, China; zhangwei0515@outlook.com (W.Z.); jiangzhiwen@ouc.edu.cn (Z.J.); chijinhua@ouc.edu.cn (J.C.); sunhuanchao@stu.ouc.edu.cn (H.S.); 21180631156@stu.ouc.edu.cn (H.L.); wanshunliu@hotmail.com (W.L.); 2 Laboratory for Marine Drugs and Bioproducts of Pilot National Laboratory for Marine Science and Technology, Qingdao 266235, China

**Keywords:** bone defect repair, butyryl chitin, hydroxyapatite, scaffold, osteogenesis

## Abstract

Bone defects, a common orthopedic problem in clinical practice, are a serious threat to human health. As alternative materials to autologous bone grafts, synthetic cell-free functionalized scaffolds have been the focus of recent research in designing scaffolds for bone tissue engineering. Butyryl chitin (BC) is a derivative of chitin (CT) with improved solubility. It has good biocompatibility, but few studies have investigated its use in bone repair. In this study, BC was successfully synthesized with a degree of substitution of 2.1. BC films were prepared using the cast film method and showed strong tensile strength (47.8 ± 4.54 N) and hydrophobicity (86.4 ± 2.46°), which was favorable for mineral deposition. An in vitro cytological assay confirmed the excellent cell attachment and cytocompatibility of the BC film; meanwhile, in vivo degradation indicated the good biocompatibility of BC. Hydroxyapatite (HA), extracted from bovine cancellous bone, had good cytocompatibility and osteogenic induction activity for the mouse osteoblast cell line MC3T3-E1. With the aim of combining the advantages of BC and HA, a BC–HA composite scaffold, with a good pore structure and mechanical strength, was prepared by physical mixing. Administered into skull defects of rats, the scaffolds showed perfect bone-binding performance and effective structural support, and significantly promoted the regeneration of new bone. These results prove that the BC–HA porous scaffold is a successful bone tissue engineering scaffold and has strong potential to be further developed as a substitute for bone transplantation.

## 1. Introduction

Bone defects are a common orthopedic problem in clinical practice, and serious injuries such as trauma, infection, and tumor resection can cause large-segment bone defects, which are a serious threat to human health [1]. Bone autograft has always been the “gold standard” material for repairing bone defects, but its clinical application is restricted due to the limited sources, secondary trauma, and pathogenic risk to the donor area [2]. Although the allograft bone have abundant sources, the risk of immune rejection and disease transmission exists [3]. As an innovative alternative for the treatment of bone defects, bone tissue engineering has been widely studied, but the application of cell-seeded scaffolds is limited due to a limited number of autologous cells, a low cell survival rate, and the high risk of immunological rejection. Therefore, cell-free functionalized scaffolds are gradually becoming a new research direction for tissue engineering scaffolds [4,5].

Chitin, the second most abundant polysaccharide in nature and comprising of 2-acetylamino-2-deoxy-D-glucopyranose units linked by β-(1-4)-glycosidic bonds, is an important component of arthropods exoskeletons [6]. Chitin has a good biocompatibility and biodegradability and shows antioxidant [7] and antibacterial activity [8]. As a wound dressing, chitin accelerates collagen deposition and promotes wound healing [9,10]. Drug carriers that are based on chitin have effectively controlled the release of anticancer drugs and reduced the side effects in humans [11]. However, the application of chitin has been limited due to its poor solubility induced by the abundant intermolecular hydrogen bonds [12]. Butyryl chitin (BC), obtained by the modification of chitin using butyrylation, improves the solubility of chitin and is easily soluble in common organic solvents, while retaining the film-forming properties of chitin. It can be used for the preparation of filaments and nonwoven fiber forms, suitable for manufacturing an assortment of BC materials for biomedical applications [13,14]. In wound healing, wound dressings prepared using BC exhibited a good biocompatibility and promoted wound healing [15,16,17]. As a drug carrier, the BC also showed good drug inclusion and release properties [18]. Although BC has excellent biological properties, there is a paucity of research investigating its application in bone regeneration.

Hydroxyapatite (HA), being the main inorganic component of bone tissues, has good biocompatibility, can enhance the activity of alkaline phosphatase (ALP) and the expression of the osteogenic gene, as well as promote osteogenic differentiation [19]. Through regulating the polarization and functional state of macrophages, HA can induce the expression of the M2 phenotype in macrophages to initiate bone formation [20]. In addition, HA has shown good osteoinduction activity in ectopic osteogenesis experiments [21,22,23]. HA has biological similarity with bone tissue and showed good effects on bone tissue repair [24,25,26]. There are an abundant of sources of natural HA, as it can be obtained from mammalian bones [27,28], fish bones, scales [29,30], shells [31,32], etc. Compared to artificial hydroxyapatite, natural HA contains more abundant trace elements [33] and better biodegradability [34], can improve the rate of bone regeneration [35], and has the advantages of being pollution-free, having a high crystallinity, and being environmentally friendly [36,37,38]. Therefore, naturally sourced HA is more suitable for biomedical applications and is an ideal inorganic component in bone repair composites. Animal-derived HA is mainly obtained using calcination of cancellous bone, which has an extremely high porosity and a large pore structure [39,40]; however, this structure also results in low mechanical strength of HA. Therefore, HA is often combined with organic substances in bone substitution materials to improve the mechanical strength [41,42,43].

In this study, BC was synthesized and its physicochemical properties and bioactivity were systematically analyzed, demonstrating that BC has good properties as a bone repair material. To further improve bone repair, HA particles were introduced into BC, and porous BC–HA scaffolds were designed and prepared. The compositional and structural properties of the scaffold were determined. The porous BCHA scaffolds were embedded in cranial defect areas in rats, their bone defect repair performance was analyzed using micro-computed tomography (Micro-CT) and histological staining, and the support strength of the scaffolds at the bone defect site was measured. These results indicate that porous BC–HA scaffolds have a good mechanical strength, could be combined with bone tissue, and effectively repair bone defects. As cell-free scaffolds, the porous BC–HA scaffolds have significant potential for bone tissue engineering.

## 2. Results and Discussion

### 2.1. Characterization of BC

**Figure 1:**
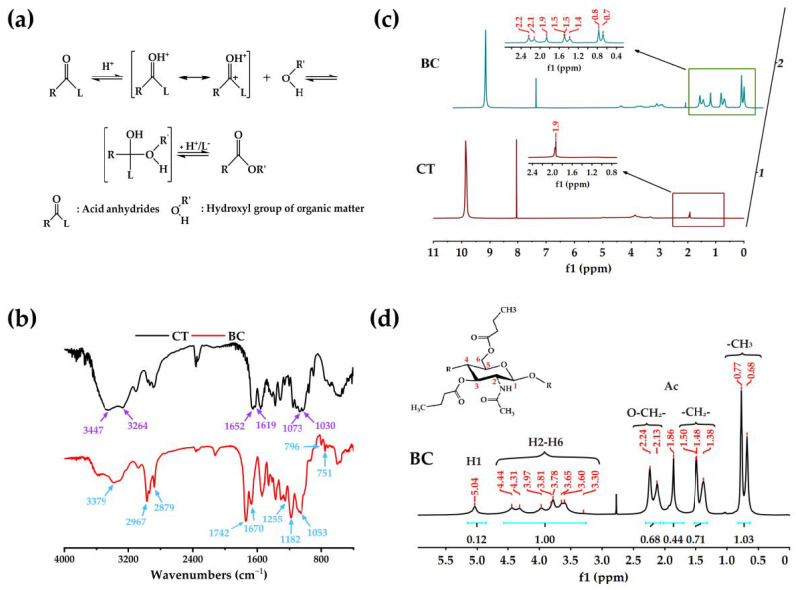
The structural characterization of chitin and BC. (**a**) The reaction mechanism of butyryl chitin synthesis. (**b**) The FTIR spectrums of chitin and BC. (**c**) The ^1^H NMR spectrums of chitin and BC. (**d**) The ^1^H NMR spectrum of BC.

The synthesis mechanism of butyryl chitin catalyzed by methanesulfonic acid is shown in Figure 1a. In this reaction, the carbonyl group of the anhydride was activated by the methanesulfonic acid, and the electrophilicity was improved. The strong hydrogen bond between molecules of chitin in the acidic system of methanesulfonic acid was opened, and the hydroxyl group as a nucleophilic group attacked the activated carbonyl carbon in the anhydride, forming a new ester bond, obtaining the butyryl chitin [44].

The Fourier transform infrared spectroscopy (FTIR) spectrum (Figure 1b) of the BC showed the appearance of additional absorption peaks of fatty acid esters at 1742 cm^−1^ (C=O), 1255 cm^−1^, and 1182 cm^−1^ (C–O), confirming the esterification of chitin. The new absorption peaks appeared around 2879–2967 cm^−1^, 796 cm^−1^, and 751 cm^−1^, which are the stretching vibration absorption peaks of methyl (–CH_3_) and methylene (–CH_2_–) in butyryl groups. The appearance of these absorption peaks indicates the successful grafting of butyryl groups into chitin. The absorption peaks of the primary alcohol hydroxyl group (C6–OH) and secondary alcohol hydroxyl group (C3–OH) of chitin at 1030 cm^−1^ and 1073 cm^−1^ were weakened and moved to 1053 cm^−1^ in BC. Additionally, the hydroxyl group (–OH) absorption peak of chitin at 3447 cm^−1^ was significantly weakened in BC, which indicates that the butyryl groups were mainly grafted onto the C-6 and C-3 hydroxyl groups of chitin. The shifting of the carbonyl absorption peak (1652 cm^−1^ and 1619 cm^−1^) toward a higher frequency (1670 cm^−1^) indicated the suppression of the hydrogen bond in chitin by the butyryl groups [13,45,46].

The hydrogen nuclear magnetic resonance (^1^H NMR) spectrum of BC is shown in Figure 1c,d with the shifts in the spectrum assigned to their corresponding protons according to the literature [45,46]. In the spectrum (Figure 1d), the acetamido methyl protons of chitin appeared at 1.86 ppm, the overlapping peaks in the range of 3.30–4.44 ppm were attributed to the H2–H6 proton signals of the chitin backbone, while the H1 proton signal of chitin appeared at 5.04 ppm. Additional signals around δ 0.68–0.77 (–CH_3_), δ 1.38–1.50 (–CH_2_–), and at δ 2.13–2.24 (O–CH_2_–) were detected. These signals further demonstrated the successful grafting of butyryl groups on chitin.

The degree of substitution (DS) of butyryl groups in BC was calculated using the combined integral methods of peak areas in the ^1^H NMR spectrum of BC (Figure 1d) according to the following Equation (1):

(1) Degree of substitution (DS)=13ICH316IH2–H6

where $I_{{\mathrm{CH}}_{3}}$ was the integral area of the methyl proton peaks (δ 0.68–0.77) of the butyryl group, and $I_{H_{2}–H_{6}}$ was the H2–H6 proton signal peaks’ (δ 3.30–4.44) integral area of the chitin backbone. Using this calculation, the DS of the BC was 2.06.

The elemental analysis results of chitin and BC are shown in Table 1. The molecular formula of chitin is expressed as [C_6_H_11_NO_4_(C_2_H_2_O)n], where n is the degree of acetylation in chitin. The molecular formula of BC is expressed as [C_6_H_11_NO_4_(C_2_H_2_O)n] (C_4_H_6_O)_DS_, where DS is the degree of substitution of the butyryl group. According to the elemental analysis results, the degree of acetyl substitution in chitin and the degree of substitution of the butyryl group in BC were calculated separately according to the Equations (2) and (3):

(2) $$\mathrm{CT}:\;{C/N}_{\mathrm{CT}}=\frac{12}{14}\times \frac{6+2n}{1}$$

(3) $$\mathrm{BC}:\;{C/N}_{\mathrm{BC}}=\frac{12}{14}\times \frac{6+2n+4\mathrm{DS}}{1}$$

Using this calculation, the n of chitin was 0.92; the DS of BC was 2.1. This result was in general agreement with the degree of substitution calculated from the ^1^H NMR spectrum integration. The prepared BC had a high degree of butyryl substitution.

The abundant intermolecular hydrogen bonding of chitin results in its poor solubility, but the substitution of hydroxyl groups by hydrophobic ester groups weakens the intermolecular hydrogen bonds of chitin and improves its solubility [46]. According to published research, BC with a high butyryl substitution degree has excellent solubility in common organic solvents [47]. In this study, the prepared BC has a high butyryl substitution degree and showed good solubility in the solubility test. It was highly soluble in all the solvents tested except ethyl acetate—especially in ethanol—which provided a good choice of solvent for the plasticity of BC.

**Figure 2:**
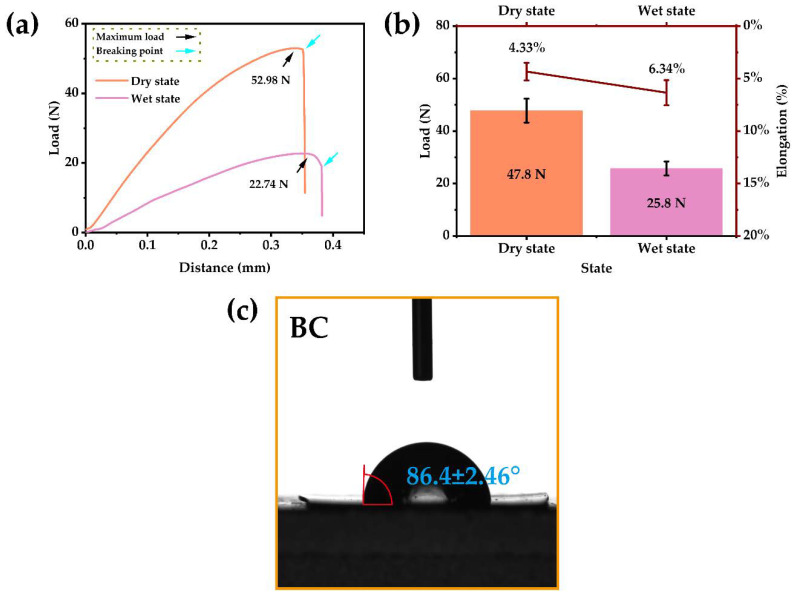
The mechanical strength and hydrophilicity of the BC film. (**a**) Representative tensile-load curves of dry and wet BC films. (**b**) The maximum load and elongation of dry and wet BC films (n = 5). (**c**) The static contact angle test result of the BC film (n = 3).

The ideal scaffold material for bone regeneration should have good mechanical strength [48], but solvents can impact the mechanical strength of the material [49]. According to the above results, ethanol is an ideal solvent, but the mechanical strength of the BC film, using ethanol as a solvent, needed to be determined. In this study, we calculated the maximum load and elongation of BC films (Figure 2a,b), in dry and wet states, to study the mechanical strength of the BC film with ethanol as the solvent. We observed that the BC film prepared with ethanol as the solvent has good mechanical strength, which further indicates that ethanol is a good plastic solvent for the BC material. However, the mechanical strength of the BC film under wet conditions (25.8 ± 2.65 N) was lower than that under dry conditions (47.8 ± 4.54 N). The mechanical strength of the BC was affected by the infiltration of moisture; therefore, we also tested the hydrophilicity of the BC films (Figure 2c). The results of the static contact angle between the BC film and deionized water (86.4 ± 2.46°) indicated that BC was weakly hydrophilic.

### 2.2. In Vitro Mineralization

**Figure 3:**
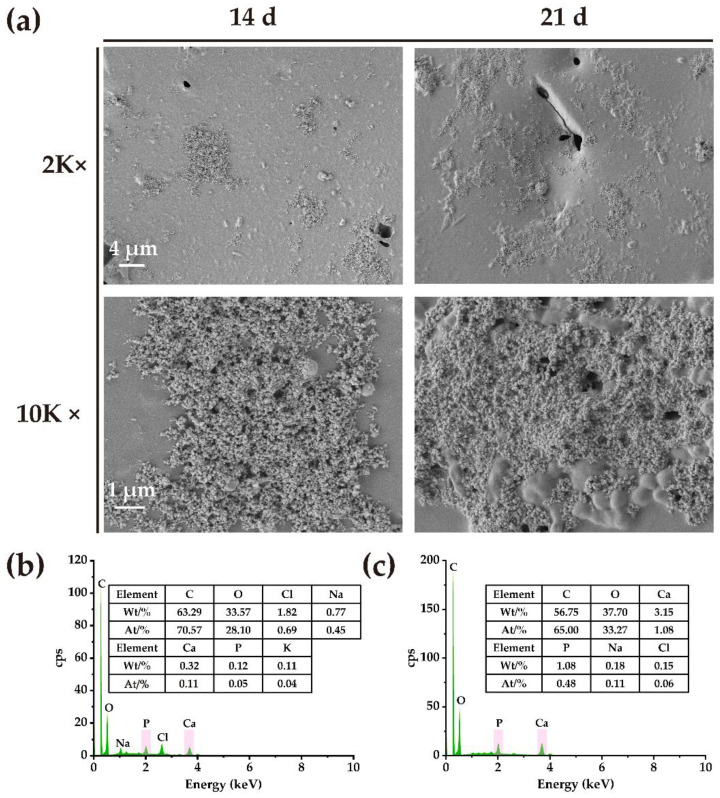
The in vitro mineralization results of the BC scaffold. (**a**) Scanning electron microscope (SEM) images of mineral deposition on the surfaces of the BC scaffolds after 14 and 21 days. (**b**) An elemental energy spectrum analysis of mineral deposition on the BC scaffold surface after 14 days. (**c**) An elemental energy spectrum analysis of mineral deposition on the BC scaffold surface after 21 days.

The ability of the material to bind to bone tissue is usually assessed by the ability of apatite to form on the surface of the material. The ion concentration of simulated body fluid (SBF) is almost equivalent to that of human plasma, and the formation of apatite on the material in SBF helps to predict the in vivo bone bioactivity of the material [50]. The formation of mineral deposition on the BC scaffold surface was observed and analyzed (Figure 3a,b). The results showed that the mineral deposition was formed after the BC was placed in SBF for 14 days, and increased after 21 days. Measurements from energy dispersive spectrometer (EDS) mapping and qualitative and quantitative analyses showed that the mineral deposition of elemental Ca and P increased significantly after 21 days compared with 14 days; the calcium-to-phosphorus ratio of mineral deposition was close to 1.67, indicating the effective in vitro mineralization ability of BC scaffolds.

As an animal exoskeleton, chitin can promote calcium carbonate deposition [51], and this in vitro mineralization ability of biomaterials is an essential characteristic reflecting its biological activity [52], as well as being an important indication of whether the material can be closely combined with bone tissue [50]. The in vitro mineralization results indicated that the BC scaffolds had a good biological activity, which should ensure its ability to bind to bone tissue.

### 2.3. Cytocompatibility of and Cell Adhesion on BC

**Figure 4:**
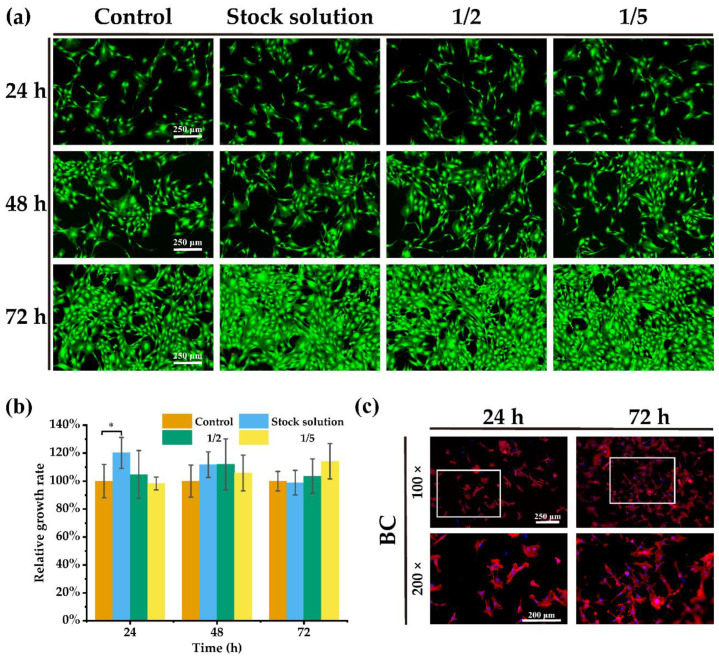
Investigation of the in vitro biocompatibility of BC films. MC3T3-E1 cells were cultured with different concentrations of the extracts from BC films incubated in culture medium. (**a**) The live/dead staining of MC3T3-E1 cells in different groups (green is living cells, red is dead cells). (**b**) The relative proliferation of MC3T3-E1 cells in different groups was analyzed using MTT (n = 3). (**c**) The cytoskeleton fluorescence staining of MC3T3-E1 cells on BC films (red is the filamentous actin of cells, blue is the nucleus of the cells). * *p* < 0.05, significant difference.

The MC3T3-E1 cell is an osteoblast cell line, which was selected here to test the cytocompatibility of BC materials and the adhesion and growth status of osteoblasts on the surface of BC film. The live and dead staining results (Figure 4a) show that the cell morphology was normal in all groups at any time point, and the number of dead cells (red) was small. The live cells (green) were evenly dispersed throughout the whole field of vision, and the number of cells increased with time. The 3-(4,5)-dimethylthiahiazo (-z-y1)-2,5-di-phenytetrazoliumromide (MTT) detection method was used to measure the cytotoxicity of the BC extraction medium. Our findings show (Figure 4b) that the relative growth rate of cells reached more than 90% in each concentration of extracts, and no significant cytotoxicity was observed compared to the control group. Therefore, the BC material had good cytocompatibility.

Cytoskeleton fluorescence staining was performed on the cells attached to the BC film to observe the state of cell adhesion (Figure 4c). The results show that, at 24 h, the cells on the film were already spreading and protruding, showing a polygonal shape, and at 72 h the cells were not only well extended, but also increased in number, which indicated that the cells could attach and proliferate normally on the BC films. The ability to support the attachment and proliferation of osteo-progenitor cells is the basis of the osteoconduction properties of certain materials [53]. The above results prove that BC not only has good cell compatibility, but also has potential as an osteoconductive material.

### 2.4. In Vivo Biocompatibility and Degradation of the BC Film

**Figure 5:**
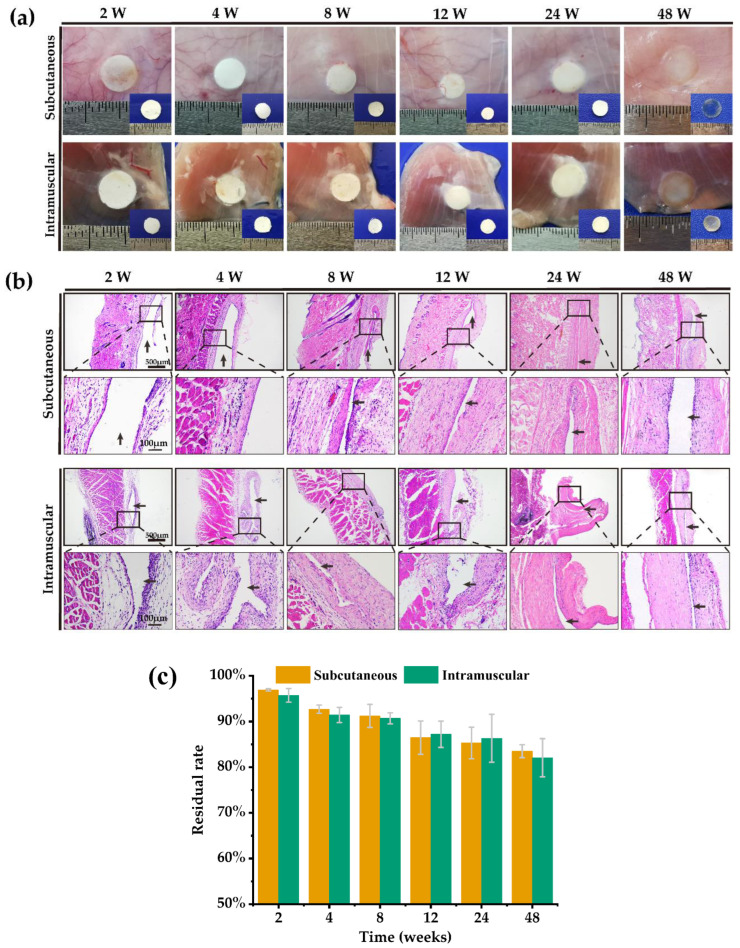
The in vivo biocompatibility and degradability of the BC film. (**a**) The in vivo biodegradation of the BC film in subcutaneous and muscle tissue at 2, 4, 8, 12, 24, and 48 weeks after surgery. (**b**) The histopathological observation of skin and muscle at the BC site (black arrows: the site of BC films). (**c**) Residual rates of subcutaneous and intramuscular BC films at 2, 4, 8, 12, 24, and 48 weeks after surgery (n = 3).

The anatomical observations of the subcutaneous and intramuscular degradation of the BC films are shown in Figure 5a. Two weeks after surgery, there was no significant organismal inflammatory reaction (such as obvious redness, oedema formation, and haemorrhage) in the tissue where the BC films were located, and the BC films were surrounded by connective tissue. As time progressed, no abnormal phenomena or obvious inflammation was observed in the tissue surrounding the BC films. The connective tissue membrane covering the BC films gradually thickened with the extension of time, and the BC films fitted more and more closely with the tissue, showing a chimeric state with the tissue at 48 weeks. Following extraction of the BC films, we found that their degradation in the subcutaneous and intramuscular areas was not significant at any time point, and the films’ morphology was still intact at 48 weeks.

The H&E staining results of skin and muscle tissue sections at the sites of the BC films are shown in Figure 5b. At 2 and 4 weeks after surgery, there was a certain degree of inflammatory cell infiltration in the skin and muscle tissues where the BC films were located, which was considered to be due to the postoperative wound healing and the normal foreign body reaction to implanted biomaterials, in our case the BC films. Foreign body reactions are the reactions of host tissue to implanted biomaterials and medical devices, which are normal immune reactions of the body and inevitable for all biomaterials [54]. After 8 weeks, the infiltration of inflammatory cells in the skin and muscle tissues disappeared, but multinucleated macrophages were seen in the connective tissue in close contact with the BC films, indicating that the BC film was undergoing a slow degradation.

The residual rates of the BC films in subcutaneous and intramuscular areas (Figure 5c) showed that the degradation rates of BC films were slow in both tissue areas, with a residual rate still above 80% at 48 weeks.

The in vivo degradation results showed that the BC film had good biocompatibility. BC is degraded in vivo mainly by hydrolysis [55], and the weak hydrophilicity observed in vitro was reflected in a slow degradation in vivo. The morphology of the BC film remaining intact in vivo for a long time showed that the porous structure of BC scaffold could be maintained in vivo without deformation.

The desirable properties of the scaffold material for bone tissue engineering include good biocompatibility, biodegradability, and mechanical durability [48]. According to the results of the present study, BC has these necessary properties, and the ability to support mineral deposition as well as cell adhesion and proliferation, which all indicate that BC is an ideal scaffold material for bone tissue engineering.

### 2.5. HA Cytocompatibility and Promotion of the Osteogenic Differentiation of MC3T3-E1 Cells

The results of live and dead staining (Figure S1a) show that the cells cocultured with different concentrations of HA extracts demonstrated normal cell morphology. The number of dead cells (red) was small, and the live cells (green) were evenly dispersed in the whole field of vision. The number of cells in the different groups increased with time. The MTT assay results show (Figure S1b) that the relative proliferation rates in the stock solution group at 24 h and 48 h were lower than in the control group, but the difference disappeared at 72 h and cell proliferation returned to normal. The other groups showed no significant differences compared to the control group at 24 and 48 h, but the relative proliferation rates in the 1/5 and 1/10 groups were higher than in the control group at 72 h and showed a highly significant difference. Our findings are similar to those of Mohammad et al. [56]. The HA extract stock solution affected the cell proliferation, but with an extension in culture time the influence gradually decreased. The low-concentration HA extract promoted cell proliferation; this suggests that the HA promotes the proliferation of osteoblast-like cells.

According to the above results, extracts (1/5, one part extract was diluted with four parts osteogenic induction medium) were selected to detect the influence of HA on the osteogenic differentiation of MC3T3-E1 cells, and the results are shown in Figure S2. Alkaline phosphatase (ALP) is a hallmark enzyme in the early stage of osteoblast differentiation and the activity it expresses is a significant feature of osteoblast differentiation [57,58,59]. According to the results of the ALP staining and activity detection (Figure S2a), the activity of ALP was slightly lower in the control group (3.53 ± 0.17 U/mg protein) than in the experimental group (3.60 ± 0.11 U/mg protein) at 7 days of osteogenesis induction, and there was no significant difference between the two groups. After 14 days of osteogenesis induction, the expression of ALP was significantly increased, and the ALP activity of the experimental group (5.63 ± 0.03 U/mg protein) was significantly higher than that of the control group (5.37 ± 0.15 U/mg protein). Alizarin red (ARS) staining and quantitative analysis results show (Figure S2b) that after 14 days of osteogenic induction, mineral depositions appeared in both the control and experimental groups and the mineralization levels were basically the same between the two groups. After 21 days of osteogenesis induction, mineralization had significantly increased in both groups, and the mineralization level in the experimental group was significantly higher than in the control group. These results indicate that HA can effectively promote the osteogenic differentiation and mineral deposition of pre-osteoblasts.

Studies have shown that hydroxyapatite can enhance cell proliferation, ALP activity, and osteogenic gene expression, and can promote osteogenic differentiation by activating the PI3K/AKT/mTOR signaling pathway [19]. ALP, an osteogenic marker expressed early during osteoblast differentiation, can provide phosphate for bone mineralization [60]. In the present study, the results of ALP and mineralization assays of MC3T3-E1 cells show that HA—prepared from bovine cancellous bone—effectively induced bone formation in osteoblasts. Osteoinductive properties are an important biological property required for bone substitutes [53]. HA is a natural material with osteoinductive activity. In order to enhance the osteo-repair effects of BC, we added HA particles to BC.

### 2.6. Characterization of the BC–HA Composite Materials

**Figure 6:**
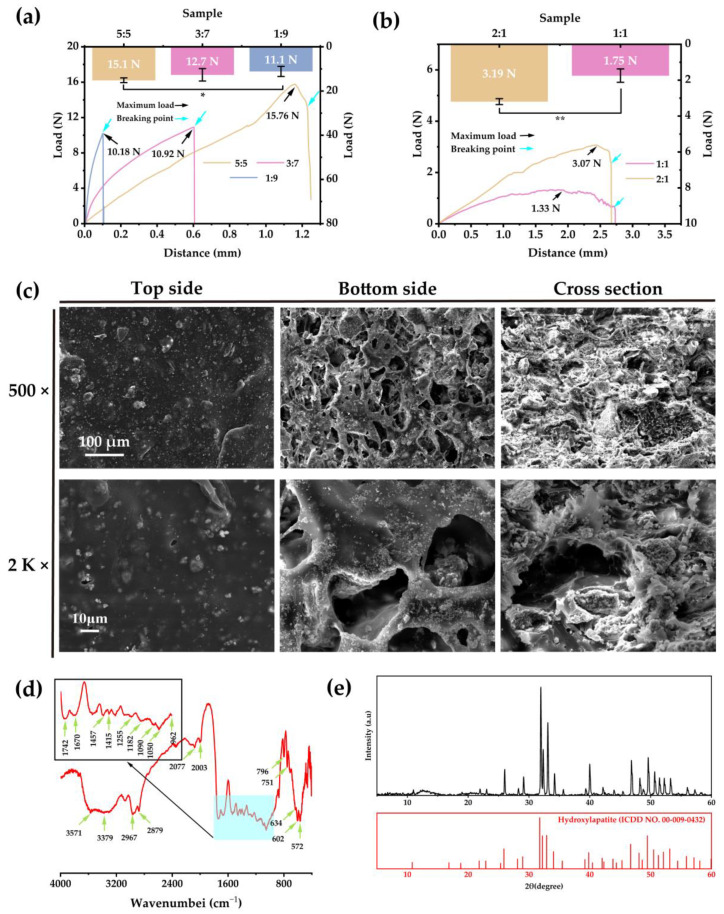
The characterization of the BC–HA composite materials. (**a**) The load–distance curves and maximum load of the BC–HA composites with different BC/HA (*w*/*w*) ratios. (**b**) The load–distance curves and maximum load of the BC–HA porous scaffolds with different BC/porogenic agent (*w*/*w*) ratios. (**c**) Representative SEM images of the BC–HA porous scaffold. (**d**) The FTIR spectrum of the BC–HA porous scaffold. (**e**) XRD patterns of the BC–HA porous scaffold. * *p* < 0.05, significant difference; ** *p* < 0.01, stronger significant difference.

The mechanical strength of the BC–HA composites was tested using the three-point bending method. The amount of HA affects the mechanical strength of the BC–HA composite (Figure 6a): an increase of HA content decreased the mechanical strength and bending moment of the composite, as well as the toughness of the material. The average load of composite materials was calculated (Figure 6a), and the results show that composite materials with a 5:5 ratio (BC:HA = 5:5, *w*/*w*) could withstand the highest maximum load (15.1 ± 1.07 N); for the 3:7 ratio (*w*/*w*), the maximum load (12.68 ± 2.82 N) was lower; and the 1:9 ratio (*w*/*w*) composites could withstand the lowest maximum load (11.13 ± 2.28 N), which was significantly different from the 5:5 ratio (*w*/*w*).

The ideal bone scaffold needs an interconnected pore structure to facilitate the inward growth of cells as well as the transport of nutrients and metabolic waste [61]. Therefore, we used the salt-leaching method to prepare the pore structure in the BC–HA composites. However, the pore structure will inevitably reduce the mechanical properties of the material [62]. Based on the above results, we chose a material composition with a 5:5 ratio as the base material for pore structure preparation. The mechanical strength of porous BC–HA scaffolds is shown in Figure 6b, which illustrates that the scaffolds with a 2:1 ratio (BC:porogenic agent = 2:1, *w*/*w*) were significantly stronger (3.19 ± 0.16 N) than those with a 1:1 ratio (*w*/*w*) (1.75 ± 0.37 N) (*p* < 0.01). Therefore, in order to create porous scaffolds with the best supporting strength, the material ratios BC: HA = 5:5 (*w*/*w*) and BC:porogenic agent = 2:1 (*w*/*w*) were the final selections to prepare the porous scaffold.

The microscopic morphology of BC–HA porous scaffolds was observed using SEM (Figure 6c). The surface of the top side of the composite scaffold has few pores, while the surface of the bottom side has an abundant porous structure. The cross-section of the composite scaffold shows that a good porous structure had been formed. The HA particles were uniformly distributed throughout the scaffold. The pore diameters in the BC–HA porous scaffold were in the range of 50–100 μm, as measured using ImageJ. Bream et al. [63] reported that new bone could form in pores smaller than 10 μm, and that the pore structures with pore diameters of less than 200 μm facilitated the formation of small vessel networks [64]. This indicates that the pore structure of the BC–HA porous scaffold is conducive to the regeneration of blood vessels and new bone.

Figure 6d shows the typical infrared characteristic peaks of porous BC–HA scaffolds. The absorption peaks of hydroxide radicals (–OH) of HA appeared at 3571 cm^−1^ and 634 cm^−1^. The unique stretching vibration absorption peaks of the O–P–O on HA appeared at 962 cm^−1^, 1050 cm^−1^, and 1090 cm^−1^; characteristic PO_4_^3-^ absorption peaks were also observed at 2003 cm^−1^ and 2077 cm^−1^. The significant stretching vibration absorption peaks of CO_3_^2-^ present in HA also appeared at 1415 cm^−1^ and 1457 cm^−1^ [30,65]. The typical infrared characteristic peaks—indicating the presence of BC—also appeared throughout the whole spectrum. The XRD pattern of the porous BC–HA scaffolds (Figure 6e) shows a significant HA crystallization peak and a non-crystallization peak of BC, at around 12°. In addition, no crystallization peak of the inorganic phases, such as CaO and CaCO_3_, was detected [66], indicating the high purity of HA.

According to the FTIR and XRD results, no new infrared absorption peaks were detected in the FTIR spectrum for the BC–HA porous scaffold, indicating that there was no chemical bond between BC and HA during the composite process, and that the two were combined in physically. The basic chemical structures of BC and HA were well preserved after the composite was formed, which ensured that the biological properties of both BC and HA were likely to be well maintained.

### 2.7. Porous BC–HA Scaffold Promoted the Repair of Skull Defects in Rats

**Figure 7:**
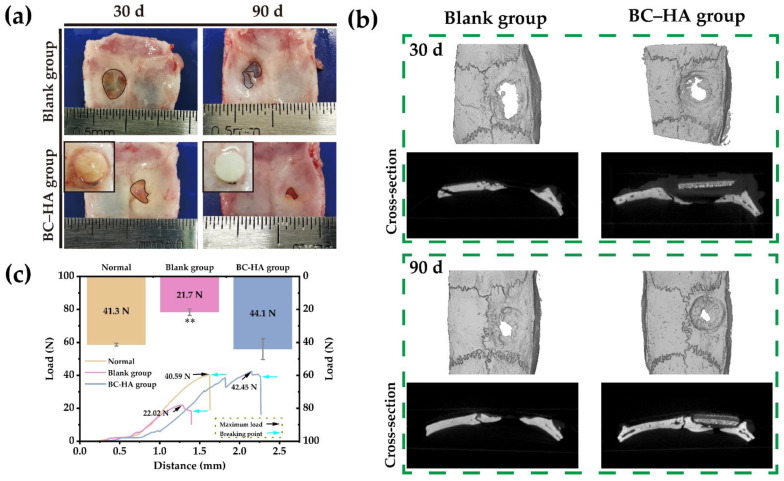
The in vivo bone defect repair performance of BC–HA composite porous scaffolds implanted in skull defects of rats. (**a**) The repair results of skull defects in the blank group and the BC–HA group after 30 and 90 days. (**b**) Representative images of three-dimensional reconstruction of the skull defect areas in the blank group and BC–HA group after 30 and 90 days. (**c**) After 90 days, the maximum load and load-displacement curves of the skull defect site in the normal group, blank group, and the BC–HA group. Compared to the normal group, ** *p* < 0.01, stronger significant difference.

The parietal calvaria critical size defect paradigm in rats is the most extensively used for evaluating the safety and efficacy of bone tissue constructs; the skull is flat and does not require plates/screws to stabilize the grafts [67]. The bone graft material can remain stable in the defect site, allowing for better observation of its integration with bone tissue. Additionally, the parietal calvaria bone osseous healing capacity is limited, requiring a long healing time [68], which makes it ideal for our slow degrading material. Therefore, the bone-repair ability of the porous BC–HA scaffold was examined by establishing a critical-size skull bone defect model in rats. The skull tissue of rats is shown in Figure 7a. In the blank group, the thin connective tissue membrane was formed at the defect site, but only a small amount of new bone was formed after 90 days, and there was still a large, unrepaired defect. In the BC–HA group, the scaffold was covered by connective tissue following implantation, and the bone defect site had a certain degree of newly regenerated bone on the 30th day. On the 90th day, the bone defect was nearly completely closed, and the scaffold was secured well on the bone defect site and closely combined with the bone tissue.

A three-dimensional reconstruction and evaluation of the cranial defect was performed using Micro-CT (Figure 7b). The results of the cranial 3D images were similar to those of the qualitative visual bone tissue observation. The blank group had less new bone formation at the defect site, while the BC–HA group had new bone formation at the edges of the defect site and extending toward the center of the defect, with the defect almost closed at 90 days.

The quantitative analysis results of the new bone at 90 days are shown in Table 2. The bone volume (BV), percent bone volume (BV/TV), and bone mineral density (BMD) of the BC–HA group were 12.75 ± 1.36 mm^3^, 41.92 ± 5.2%, and 0.27 ± 0.02 g/cm^3^, respectively, which were higher than those of the blank group (7.36 ± 1.01 mm^3^, 24.14 ± 3.12%, and 0.10 ± 0.01 g/cm^3^). The area of the residual bone defect in the BC–HA group was only 2.03 ± 0.68 mm^2^, which was significantly lower than that of the blank group (3.8 ± 0.27 mm^2^). These findings suggest that the BC–HA scaffold can promote the regeneration of new bone at the defect site.

In cranial defect repair, the repair material needs to have mechanical strength in order to provide support [69]. The mechanical strength of cranial bone tissue of rats at 90 days was tested using three-point bending (Figure 7c). The mean maximum load of the blank group was 21.73 ± 1.99 N, which was significantly lower than that of the normal skull (41.34 ± 0.83 N), while the mean maximum load of the BC–HA group (carrying material) (44.08 ± 6.3 N) was similar to that of the normal skull, with no significant difference. This indicates that BC–HA scaffold could not only promote the repair of cranial defects, but also that the overall load-bearing strength of the scaffold and cranial bone was similar to that of normal cranial bone after 90 days of bone tissue repair and could play a supporting role at the bone defect site.

**Figure 8:**
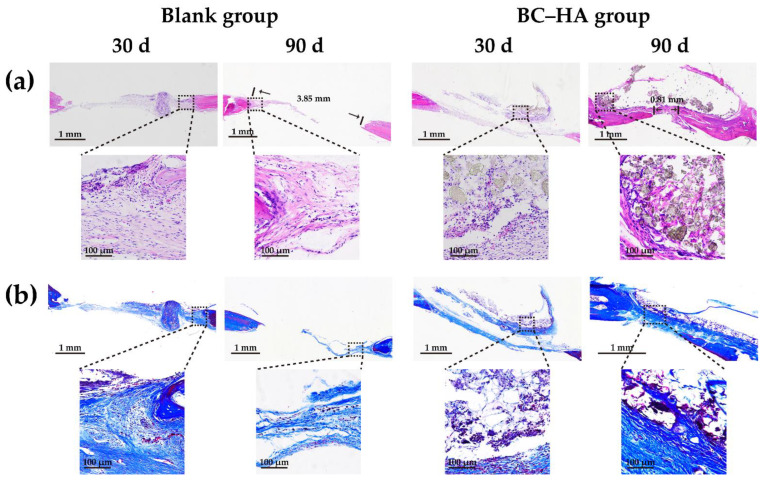
Histomorphological observations of bone defect areas on stained tissue sections. (**a**) H&E staining and (**b**) Masson’s trichrome staining.

The H&E and Masson’s trichrome staining results of the cranial tissue sections (Figure 8a,b) were consistent with the Micro-CT results, and successful new bone regeneration was seen in the BC–HA group. The defect site in the blank group was covered by a thin layer of fibrous connective tissue, while the scaffold in the BC–HA group was covered by collagen fibrous tissue. The collagen fibers grew into the BC–HA scaffold and formed a mesh-like structure wrapped around the HA particles. This not only increased the stability of the scaffold at the bone defect site, but also promoted the regeneration of new bone through the osteogenic induction provided by HA. The results of H&E and Masson staining further indicated that the porous BC–HA scaffolds could closely bond with bone tissue, and could effectively promote the reconstruction and regeneration of new bone at the bone defect site.

According to the above results, the porous BC–HA composite scaffold presented a good bone repair effect in the repair of skull defects in rats; however, as a non-load-bearing bone, the cranial defect model of rats has certain limitations. Therefore, in order to better test the osteogenic and mechanical properties of the composite scaffold, load-bearing bone defect models (such as tibial defect [70], femur defect [71], and ulna defect [72], etc.) should be selected for further verification. For fracture fixation implants, mechanical properties need to be maintained for 3–6 months throughout the treatment cycle [72]. The mechanical properties of the material are affected by degradation and erosion after implantation, so further analysis of the changes in the mechanical strength of the BC–HA composite scaffold in vivo is required.

## 3. Materials and Methods

### 3.1. Preparation and Characterization of Butyryl Chitin (BC)

#### 3.1.1. Preparation of BC

Using methanesulfonic acid as a solvent and catalyst, according to the material ratio, the butyric anhydride and methanesulfonic acid were mixed using a 0 °C water bath. Following homogeneous mixing, the chitin (acetyl degree of 92%, Qingdao Biotemed Biomaterial Co., Ltd., Qingdao, China) was added according to the material ratio and stirred for 12 h at 30–40 rpm. At the completion of the reaction, the products were neutralized using a 1 M NaHCO_3_ solution, washed with deionized water, and oven dried overnight to obtain the BC.

#### 3.1.2. Characterization of the Chemical Structure of BC

The chemical structure of BC was analyzed using FTIR. The BC material was dried at 60 °C for 24 h. A small amount of BC material was evenly mixed with KBr particles and ground into a fine powder, then placed in the mold and pressed into a tablet. A Fourier infrared spectrometer (Avatar360, Thermo Nicolet, Madison, WI, USA) was used for spectral scanning in the range of 400–4000 cm^−1^.

Deuterated formic acid-D_2_ (D, 98%) (<5% D_2_O) (Cambridge Isotope Laboratories, Inc., Tewksbury, MA, USA) was used as the deuterium solvent. The dried BC (10 mg) was dissolved in 500 μL of deuterated formic acid and transferred into an NMR tube, after complete dissolution at room temperature. The ^1^H NMR of the BC was obtained using an NMR spectrometer (VANCE III600, Bruker, Karlsruhe, Germany). The NMR spectrum was analyzed using MestReNova software (14.2.0).

The BC material was fully dried at 60 °C and the C, H, and N contents were determined using elemental analysis (FLASH EA1112, Thermo Electron SPA, Waltham, MA, USA).

#### 3.1.3. Solubility of BC

The solubility of BC in different organic solvents was tested as follows: the BC was dried overnight at 60 °C, and the material was accurately weighed (0.02 g) and added into 2 mL of different organic solvents (formic acid, pyridine, dichloromethane, tetrahydrofuran, acetonitrile, dimethyl sulfoxide (DMSO), ethanol, ethyl acetate, and acetone), shaken thoroughly, and the dissolution state of the samples was observed.

#### 3.1.4. Mechanical Strength of BC Films

In order to determine the strength of the BC film, 0.6 g of BC was dissolved in 10 mL of ethanol and a 20 mm × 10 mm × 1 mm film was prepared using the cast film method [73]. The tensile strength of BC films, in dry and wet states, was measured using an electronic universal testing machine (AGS-X, SHIMADZU, Tokyo, Japan). To achieve the wet state, films were immersed in PBS (pH = 7.4) for 24 h at room temperature before being tested. The drawing speed was set to 1 mm/min and the gauge distance was set to 10 mm. The load and elongation at breaking point were recorded.

#### 3.1.5. Hydrophilicity of BC Films

The static contact angle of the BC film was measured using a surface tensiometer (K100, KRUSS, Hamburg, Germany) using the sessile drop method. Deionized water (8 μL) was gently placed on the surface of the BC film, photographed, and measurements were recorded after 30 s. Three areas of each film were randomly selected for measurement, and the shape of the droplets was fitted using the static drop model (KRÜSS ADVANCE1.13.2.06901).

#### 3.1.6. In Vitro Mineralization

Porous BC scaffolds, with a diameter of 5 mm and thickness of 1 mm, were prepared using the salt-leaching method and sterilized with ^60^Co. The sterile porous BC scaffolds were immersed in 15 mL of simulated body fluid (SBF, CB3160, G-CLONE, Beijing, China) in a polyethylene bottle at 37 °C for 14 and 21 days. The mineralized results were observed using SEM (VEGA3, TESCAN, Brno, Czech Republic) and the elemental content of mineral deposits was analyzed using energy-dispersive X-ray spectroscopy (EDS) (VEGA3, TESCAN, Brno, Czech Republic).

#### 3.1.7. Cytocompatibility Assays of the BC Films

BC films measuring 20 mm × 10 mm × 2 mm and BC circular films with a 5 mm diameter were prepared and sterilized with ^60^Co for subsequent cell experiments. The sterile BC films were immersed in a α-MEM complete medium (α-MEM, 12000063, Thermo Fisher, Waltham, MA, USA) containing a 100 U/mL penicillin–streptomycin solution (P1400, Solarbio, Beijing, China) and a 10% fetal bovine serum (FBS, 04-001-1 A, Biological Industries, Beit-HaEmek, Israel), with a 0.1 g/mL ratio, and incubated for 72 h at 37 °C. Subsequently, the extraction medium was sterilized with a 0.22-μm filter and diluted with a complete medium at 1-fold and 4-fold dilution for the cytocompatibility assays (stock solution, 1/2 extraction medium, and 1/5 extraction medium). A mouse osteoblast cell line (MC3T3-E1) was purchased from the Kunming Cell Bank of the Chinese Academy of Science (Kunming, China).

Cell proliferation and morphological assessment: Suspensions of MC3T3-E1 cells at the logarithmic growth stage were prepared with a density of 1 × 10^4^ cells/mL 200 μL/well added into the wells of 96-well plates, and the samples were incubated in a humid atmosphere containing 5% CO_2_ at 37 °C. After 24 h of cell adhesion, the cell medium of the experimental groups was replaced with the BC extraction medium (stock solution, 1/2 extraction medium, and 1/5 extraction medium), the control group medium was replaced with fresh complete medium, and the wells for the blank group received cell-free medium (200 μL/well). Following further cell incubation for 24, 48, and 72 h, the live/dead cell staining kit (C2015S, Beyotime, Shanghai, China) was used for staining the live/dead cells. The distribution and morphology of the cells were observed using a fluorescence microscope (Eclipse Ts2R-FL, Nikon, Tokyo, Japan). The MTT (M2128, Sigma, St. Louis, MO, USA) method [44] was used to detect the metabolic efficiency of the cells as an index of their viability. This experiment was repeated three times and the mean value was calculated.

Cell adhesion assay: The sterile BC circular films were placed at the bottom of wells of 96-well plates, and MC3T3-E1 cells were added. The suspensions of MC3T3-E1 cells at the logarithmic growth stage were prepared with a density of 1 × 10^4^ cells/mL and added to the 96-well plates at 200 μL/well. Cells were cultured in a humid atmosphere containing 5% CO_2_ at 37 °C. After incubation for 24 and 72 h, the cytoskeleton and nuclei were stained using the actin-tracker Red-Rhodamine (C2207S, Beyotime, Shanghai, China) kit, and the morphology of the cells on the surface of the BC film was observed using a fluorescence microscope (Eclipse Ts2R-FL, Nikon, Tokyo, Japan).

#### 3.1.8. In Vivo Degradation of BC Films

Male SD rats (220 ± 20 g) were selected as the experimental model. The BC films with a 5 mm diameter were prepared according to the aforementioned method and sterilized with ^60^Co for the following assay.

The biodegradability of BC films was investigated by implanting BC films into the subcutaneous and intramuscular tissues of SD rats. The initial weight of the BC films was recorded before implantation. The sterile BC films was implanted in the subcutaneous tissue at the back and in the leg muscles of the SD rats. Three rats were sacrificed at each of 2, 4, 8, 12, 24, and 48 weeks after surgery. The residual BC films were collected. Following cleaning using deionized water and drying in the oven, the residual BC films were weighed. The residual rate of the BC material was calculated according to the following equation:

(4) $$Residual\;rate\;\left(\%\right)=\frac{W_{t}}{W_{o}}\times 100\%$$

where *W_t_* is the weight of residual BC films at different time points and *W_o_* is the weight of BC films before implantation. The tissue from the implantation site was fixed with 4% paraformaldehyde, embedded using paraffine, and the sections with 5 μm were stained with matoxylin and eosin (H&E) to observe tissue inflammation.

### 3.2. Preparation and Characterization of BC–HA Composite Materials

#### 3.2.1. Preparation of BC–HA Composite Scaffolds

The BC was dissolved in ethanol at a concentration of 6% and the HA particles (from bovine femur cancellous bone sieved using a 400-mesh sieve) were added according to the mass ratio of BC/HA (1:9, 3:7, 5:5). The components were evenly mixed and placed in a rectangular mold. Following the complete volatilization of the solvent, the composite material was immersed in deionized water and cleaned repeatedly to remove the residual solvent. Following drying, the composite materials were cut into 20 mm × 10 mm × 2 mm rectangular plates for subsequent analyses.

The porous BC–HA composite scaffolds were prepared using the salt-leaching method. Briefly, the BC was dissolved in ethanol at a concentration of 6% and the HA particles were added according to the mass ratio of BC/HA (5:5); meanwhile, the porogenic agent (NaCl particles, sieved using a 400-mesh sieve) was added according to the mass ratio of the BC/porogenic agent (2:1 and 1:1). The mixture was combined evenly and placed into a rectangular mold. Following the volatilization of the solvent, the composite material was immersed in deionized water for 24 h. Following drying, the film plate was cut into a 20 mm × 10 mm × 2 mm rectangular plate for subsequent detection.

#### 3.2.2. Mechanical Strength of BC–HA Composite Materials

The mechanical strength of BC–HA composite films and porous BC–HA composite scaffolds was tested using an electronic universal testing machine (AGS-X, SHIMADZU, Tokyo, Japan) using the three-point bending test. The lower span was set at 10 mm and the loading speed was set at 5 mm/min to detect the load on the film.

#### 3.2.3. SEM imaging of Porous BC–HA Composite Scaffolds

The BC–HA composite porous scaffolds were bonded to a conductive adhesive. Gold was sprayed onto the surface of the materials to achieve conductivity. The top, bottom, and a cross-section of the materials were imaged using a scanning electron microscope (SEM) (VEGA3, TESCAN, Brno, Czech Republic) to assess the morphology of the materials.

#### 3.2.4. FTIR Spectroscopy and XRD of Porous BC–HA Composite Scaffolds

The composition of the BC–HA composite scaffolds was analyzed using FTIR. The BC–HA composite scaffolds were fully dried at 60 °C. A small amount of material was evenly mixed with KBr particles and ground into a fine powder, then placed in a mold and pressed into a tablet. A Fourier infrared spectrometer (Avatar360, Thermo Nicolet, Madison, WI, USA) was used for spectral scanning in the range of 400–4000 cm^−1^.

The BC–HA composite material was also analyzed using XRD (D8 ADVANCE, Bruker, Karlsruhe, Germany), with a CuKα ray source, a 0.154 nm wavelength, a 40 KV/30 mA test voltage and current, and a test angle range of 5–90°.

### 3.3. Induction of In Vivo Bone Repair by Porous BC–HA Composite Scaffolds

Male SD rats (220 ± 20 g) were selected for the experiment. All procedures performed on the animals were approved by the Ethics Committee of Ocean University of China (OUC-AE-2022-133) and in accordance with the guidelines of the National Institutes of Health on the use and care of laboratory animals. Porous BC–HA composite scaffolds with a 5-mm diameter and a thickness of 1 mm were prepared according to the aforementioned method and sterilized with ^60^Co for the following assay.

The rat skull-defect model was selected to study the bone repair effect of composite scaffolds. Briefly, each rat was general anesthetized with 3% pentobarbital sodium, a 5 mm diameter hollow drill was used to create 5 mm diameter full-layered cranial defects in the parietal bone areas of rats. The rats with cranial defects were randomly divided into the BC–HA group and the blank group, with 10 rats per group. The sterile BC–HA composite porous scaffolds were implanted in the bone defect areas in the BC–HA group, and no material was added to the bone defect areas in the blank group. Penicillin was injected intramuscularly for 7 consecutive days after surgery to prevent wound infection.

At 30 and 90 days post-operation, the SD rats were sacrificed by means of overdose anesthesia, and skull samples were collected. The skulls were photographed, and bone tissue samples were fixed with 10% paraformaldehyde and stored in PBS at 4 °C. The bone samples were scanned using a Micro-CT (Bruker Skyscan 1276, Karlsruhe, Germany) with a resolution of 10 μm, and 3D images were reconstructed, allowing for the calculation of the bone volume (BV), percentage of bone volume (BV/TV), minimum defect area, and bone mineral density (BMD) of the defect site at 90 days. In order to determine the supporting strength of the scaffolds, the three-point bending mechanical test (AGS-X, SHIMADZU, Tokyo, Japan) was performed on the samples (the skulls that contained scaffolds) at 90 days. Subsequently, the samples were decalcified using a 10% (*w*/*v*) ethylene diamine tetraacetic acid (EDTA) solution at room temperature for 4 weeks. Following this, the samples were dehydrated, embedded using paraffin, and sections with 5 μm were stained with H&E and Masson’s trichrome (Solarbio, Beijing, China) according to the kit instructions. The state of new bone regeneration at bone defect sites were observed and photographed using an optical microscope (Eclipse Ci-L, Nikon, Tokyo, Japan).

### 3.4. Statistical Analysis

All experiments were independently repeated at least three times. The results are expressed as mean ± standard deviation. Statistical analyses were performed using IBM SPSS statistical software (V26.0). One-way analysis of variance (ANOVA) was performed, followed by the Least-Significant Difference (LSD) test for multiple comparisons (* *p* < 0.05, ** *p* < 0.01).

## 4. Conclusions

In conclusion, BC was successfully synthesized with a DS of 2.1, and dissolved well in ethanol with excellent mechanical strength. The BC had good cytocompatibility, could promote mineral deposition, and supported cell adhesion and proliferation on its surface. The in vivo degradation test proved that BC had good biocompatibility and a slow degradation rate and could maintain a certain morphology in vivo for a considerable time without deformation. These results indicate that BC had good properties as a bone repair material. HA had good cytocompatibility and could significantly promote the osteogenic differentiation of MC3T3-E1 preosteoblastic cell line in vitro. In order to enhance the effect of BC on bone repair, it was combined with bovine bone-derived HA to create porous BC–HA scaffolds, which proved good mechanical strength. The SEM images showed that the porous BC–HA composite scaffolds had a good pore structure, and FTIR and XRD analyses further confirmed that the BC and HA were physically mixed. The in vivo cranial defect repair results demonstrated that the porous BC–HA scaffolds could significantly promote bone tissue regeneration in cranial defects in rats, while being able to merge with the surrounding bone tissue and play a supportive role. Our results indicate that the porous BC–HA scaffolds integrated the positive properties of both the BC and HA. As an effective composite porous scaffold with a reliable mechanical strength, porous BC–HA scaffolds can be further developed and, in the future, be applied as tissue engineering scaffolds for bone tissue repair. The scaffolds developed in this study have significant potential for the development as an alternative to bone grafts.

## Figures and Tables

**Table 1:** The elemental analysis results of chitin and BC.

Sample	C (%)	H (%)	N (%)	C/N
CT	39.03%	4.19%	5.80%	6.72
BC	54.64%	5.92%	3.90%	14.01

**Table 2:** The bone tissue parameters of the cranial defect area in the BC–HA and blank groups at 90 days post-treatment.

90 Days	Bone Volume (mm^3^)	Percent Bone Volume (BV/TV, %)	Minimum Defect Area (mm^2^)	Bone Mineral Density (BMD g/cm^3^)
Blank	7.36 ± 1.01	24.14 ± 3.12	3.8 ± 0.27	0.10 ± 0.01
BC-HA	12.75 ± 1.36 **	41.92 ± 5.2 **	2.03 ± 0.68 *	0.27 ± 0.02 **

* *p* < 0.05 significant difference; ** *p* < 0.01 stronger significant difference.

## Data Availability

The data used in this work are available from the first authors or the corresponding authors.

## Supplementary Materials

The following supporting information can be downloaded at: https://www.mdpi.com/article/10.3390/ijms24108519/s1.
