# Supplementary material for: A Novel Porous Butyryl Chitin–Animal Derived Hydroxyapatite Composite Scaffold for Cranial Bone Defect Repair
Source: Int J Mol Sci. 2023 May 10;24(10):8519. doi: 10.3390/ijms24108519 (PMC10217846; doi:10.3390/ijms24108519)
Supplement: Supplementary file 1 [file ijms-24-08519-s001.zip › ijms-2377029-supplementary.pdf]

Supplementary materials

Results

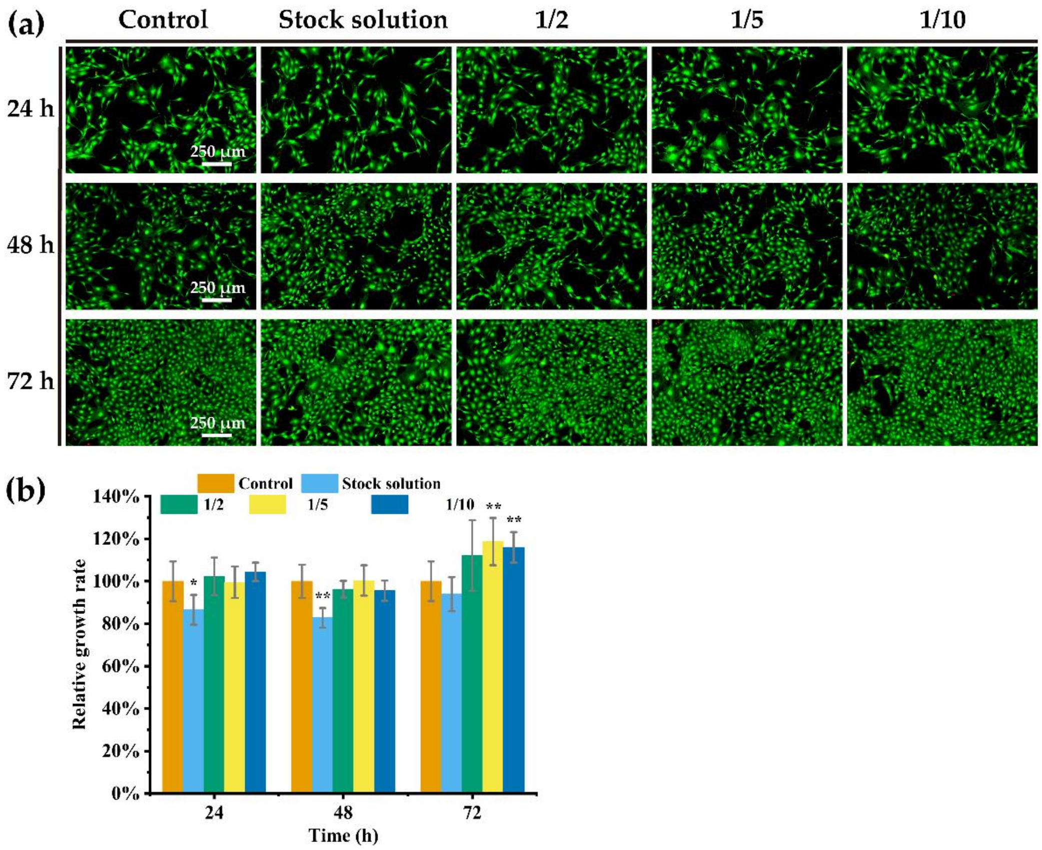

Figure S1. The in vitro biocompatibility investigation of HA. MC3T3-E1 cells were cultured with HA culture-medium extracts of different concentrations. **(a)** The live/dead staining of MC3T3-E1 cells in different groups. **(b)** The relative proliferation of MC3T3-E1 cells in different groups was analyzed using MTT (n=3). $*P < 0.05$, significant difference, $**P < 0.01$, stronger significant difference.

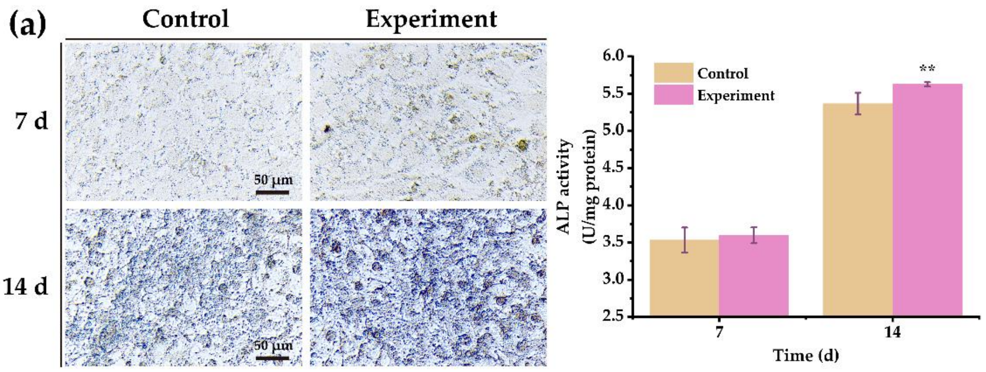

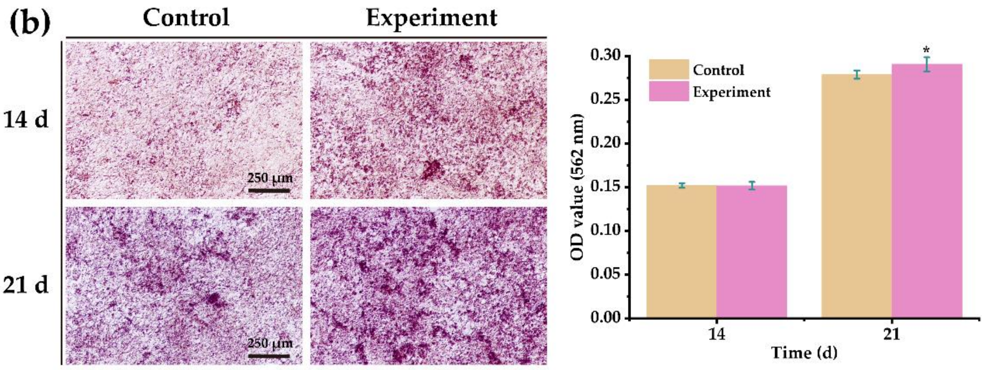

Figure S2. The in vitro osteogenesis properties of HA. **(a)** Representative images of alkaline phosphatase (ALP) staining after 7 d and 14 d of osteogenic induction and ALP activity in different groups. **(b)** Representative images of alizarin red S (ARS) staining after 14 d and 21 d of osteogenic induction and quantitative results of ARS staining in different groups. *$P < 0.05$, significant difference, **$P < 0.01$, stronger significant difference.

Materials and Method

Determination of the cytocompatibility and osteogenic activity of HA

The main component of bovine calcined bone prepared in our laboratory was identified as hydroxyapatite (HA). Sterilized HA particles were immersed in complete α-MEM containing 100 U/mL penicillin-streptomycin solution and 10% fetal bovine serum with a ratio of 0.1 g/mL, incubated at 37°C for 72 h to obtain HA extraction medium. Subsequently the extraction medium was sterilized using 0.22 μm filters and diluted with complete medium at 1-fold, 4-fold, and 9-fold dilution for the cytocompatibility assays. In addition, 1 g of sterilized HA particles was immersed in osteogenic induction medium (complete α-MEM, 50 μg/mL ascorbic acid, and 10 mM β-sodium glycerophosphate) at 37°C for 72 h. Following this, the extraction medium was sterilized with a 0.22-μm filter and diluted with osteogenic induction medium at 4-fold dilution for osteogenic activity assays.

Cell proliferation and morphology assessment

Suspensions of MC3T3-E1 cells at the logarithmic growth stage were prepared with a density of 1×10^4 cells/mL and added to the 96-well plates at 200 μL/well, and incubated in a humid atmosphere containing 5% CO_2 at 37°C. Following 24 hours of cell adhesion, the cell medium of the experimental groups was replaced with the extraction medium from HA particles (the undiluted stock solution,1/2 extraction medium, 1/5 extraction medium, and 1/10 extraction medium). The control group received fresh complete medium, and the blank group received cell-free medium (200 μL per well). Following cell incubation for 24, 48, and 72 h, the live/dead cell staining kit was used for live/dead cells. The distribution and morphology of the cells were observed using a fluorescence microscope (Eclipse Ts2R-FL, Nikon, Tokyo, Japan).

The MTT method was used to determine the relative growth and proliferation of cells. This experiment was repeated three times and the mean value was calculated.

Alkaline phosphatase (ALP) activity assessment

Suspensions of MC3T3-E1 cells at the logarithmic growth stage were prepared with a density of 1×10^4 cells/mL, added to 12-well plates at 1 mL/well, and incubated in a humid atmosphere containing 5% CO_2 at 37°C. After 24 hours of cell adhesion, the cell medium of the experimental groups was replaced with the extraction induction medium from HA particles (1/5, one part extract was diluted with four parts osteogenic induction medium), and the control group was replaced with fresh osteogenic induction medium. The culture medium was changed every 3 days. Following the induction of osteogenesis for 7 and 14 days, ALP staining of the cells was performed using the Alkaline Phosphatase Staining kit (G1480, Solarbio, Beijing, China). Additionally, the cellular proteins were extracted and the ALP activity was determined using an ALP assay kit (P031S, Beyotime, Shanghai, China), and the protein concentration was tested using a BCA kit (P0012S, Beyotime, Shanghai, China). The ALP activity was expressed as U/mg protein.

Determination of mineral deposition by cells

Suspensions of MC3T3-E1 cells at the logarithmic growth stage were prepared with a density of 1×10^4 cells/mL, added to 6-well plates at 2 mL/well, and incubated in a humid atmosphere containing 5% CO_2 at 37°C. After 24 hours of cell adhesion, the cell medium of the experimental groups was replaced with the HA particle extraction induction medium (1/5, one part extract was diluted with four parts osteogenic induction medium), and the control group received fresh osteogenic induction medium. The culture medium was changed every 3 days. Following the induction of osteogenesis for 14 and 21 days, the cells were stained with 0.2% (w/v) alizarin red dye and photographed microscopically (Eclipse Ci-L, Nikon, Tokyo, Japan). Following this, the alizarin red dye was extracted using 10%(w/v) cetylpyridine chloride at 37℃ for 1 h. The absorbance of the solution at 562 nm was determined using a microplate reader (Multiskan Go 1510, Thermo Fisher Scientific, Wisconsin, USA), and compared the mineral deposition content between control and experimental groups according to the OD value.
